# Becoming the Standard: Expanding the Conventional Use of Interventional Embolization for Ruptured Pulmonary Artery Aneurysms

**DOI:** 10.7759/cureus.3576

**Published:** 2018-11-12

**Authors:** Taylor S Harmon, Guneet Kaleka, Lynsey M Maciolek, Manoj K Kathuria, Gunvir S Gill, Aun Hussain, Stephen Dooley, Arya N Bagherpour

**Affiliations:** 1 Interventional Radiology, University of Texas Medical Branch, Galveston, USA; 2 Radiology, Oakland University William Beaumont School of Medicine, Southfield, USA; 3 Radiology, University of Texas Medical Branch, Galveston, USA

**Keywords:** behcet’s disease, vasculitis, embolization, pulmonary artery aneurysm, interventional radiology, coils, tuberculosis, endovascular, sandwich technique, hemorrhage

## Abstract

Behcet’s disease is inflammatory vasculitis that has a high incidence of mortality in patients with pulmonary artery aneurysm (PAA) formation. Traditionally, patients with Behcet’s disease and PAA rupture undergo invasive surgical management. Surgical intervention; however, has been shown to have high complication, failure, and mortality rates. It has become a more contemporary practice to utilize the interventional embolization of pulmonary artery aneurysms (PAAs) in patients with Behcet’s disease and other various etiologies because of its inherent minimally invasive nature and decreased risk for complications. The management paradigm for treating PAAs has shifted toward endovascular embolization even in severe or emergent cases where surgical management was once thought to be the standard. The following case is a testimony to the practicality of interventional embolization in the setting of a symptomatic patient presenting with PAAs.

## Introduction

Behcet’s disease mostly causes venous occlusion and varix formation but a pulmonary arterial aneurysm rupture is the highest cause of mortality in these patients [1-2]. The first Behcet’s disease-associated pulmonary artery aneurysm (PAA) was documented in 1959, with the management of its consequences evolving since then [2]. Currently, it is reported that 10% of patients with Behcet’s disease have pulmonary artery aneurysms (PAAs) that usually present profusely throughout the pulmonary arterial vasculature [3]. Though Behcet’s disease does not discriminate by gender, it is known to have a more severe disease occurrence in young men below 25 years of age [2]. Furthermore, other disease processes, such as structural cardiac anomalies, structural vascular anomalies, pulmonary hypertension, and infection, are known to cause PAAs [4]. If patients with Behcet’s disease have any of the codominant aforementioned disease etiologies, it is predicted that an even higher risk of PAA and subsequent rupture is likely.

The surgical management of a PAA rupture in patients with Behcet’s disease has historically been the standard of care, resulting in partial or total lobectomy [5]. However, because of the invasive nature of surgical management and severe complications, such as hepatic failure leading to patient death, endovascular embolization is emerging as a conventional method for PAAs in patients with Behcet’s disease [5]. Moreover, other factors of patient presentation may have an effect on the management of PAAs, prospectively leading to interventional management and the avoidance of invasive surgery altogether. Though surgical management is still clearly indicated in certain situations, the endovascular embolization of PAAs has quickly expanded in conventional use across a conglomerate of disease etiologies and patient presentations. The following case presents a symptomatic, hemodynamically stable, young male with Behcet’s disease and concurrent tuberculosis, who received endovascular embolization of PAAs.

## Case presentation

**Figure 1:**
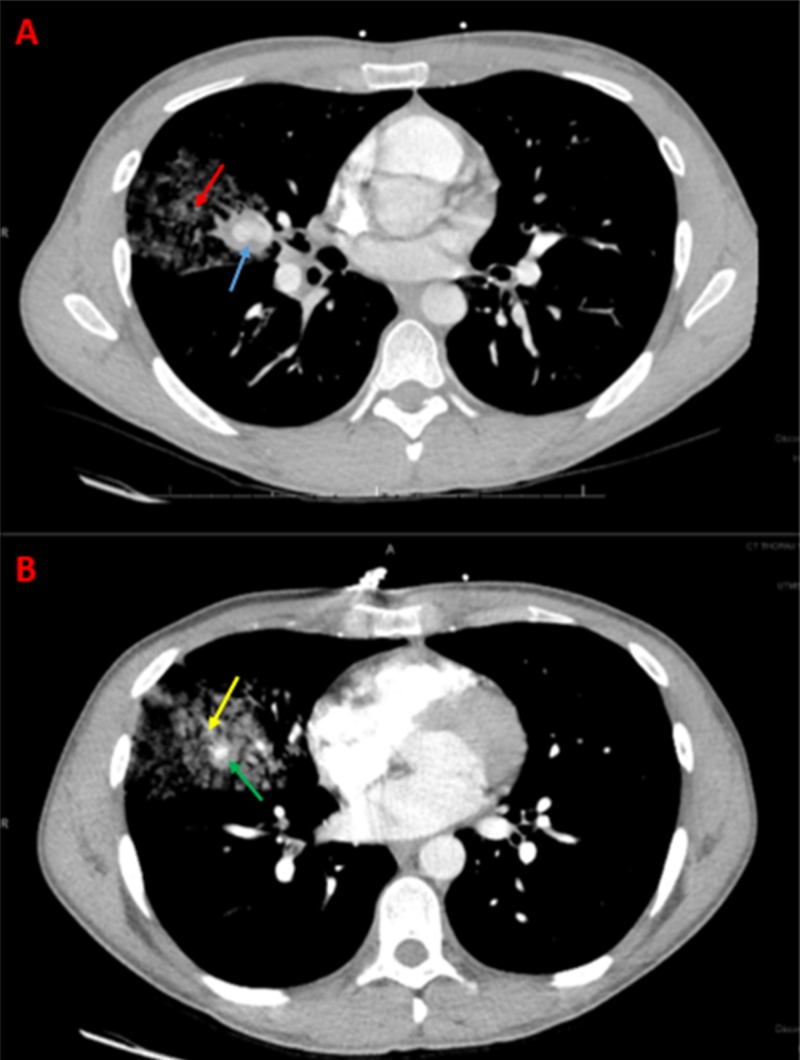
Axial Computed Tomography Angiogram Demonstrating Pulmonary Artery Aneurysms and Respective Hemorrhage An axial computed tomography angiogram of the chest demonstrates two separate pulmonary artery aneurysms in the right middle lobe associated with a surrounding pulmonary artery hemorrhage. The top frame (A) shows the most proximal aneurysm (blue arrow) with the respective hemorrhage (red arrow). The bottom frame (B) shows a more distal aneurysm (green arrow) with the respective hemorrhage (yellow arrow).

A 27-year-old African American male, with a past medical history of aphthous and genital ulcers, tuberculous meningitis, brainstem encephalitis, and transverse sinus thrombosis, presented with the onset of sudden massive hemoptysis. Computed tomography angiography (CTA) of the chest demonstrated two, separate pulmonary artery aneurysms in the right middle lobe, associated with a surrounding pulmonary artery hemorrhage (Figure 1).

The patient also had acute pulmonary embolisms in the bilateral upper lobes of the lungs. Given the patient’s constellation of symptoms, he was diagnosed with Behcet’s disease. After a multidisciplinary discussion with the intensive care unit and thoracic surgery teams, the decision was made for the patient to receive a pulmonary angiogram with the embolization of the aneurysms and the placement of an inferior vena cava (IVC) filter.

After accessing the right common femoral vein using ultrasound guidance, a seven French, 55 cm guiding sheath was placed into the main pulmonary artery under fluoroscopic guidance. Through the sheath, a five French pigtail catheter was advanced into the main pulmonary artery. A subsequent pulmonary angiogram was performed, which was grossly unremarkable. The pigtail catheter was exchanged for a four French glide catheter, which was then advanced into the right main pulmonary artery. An angiogram was then performed, which demonstrated an aneurysm filling supplied by the lateral pulmonary arterial segment of the right middle lobe.

**Figure 2:**
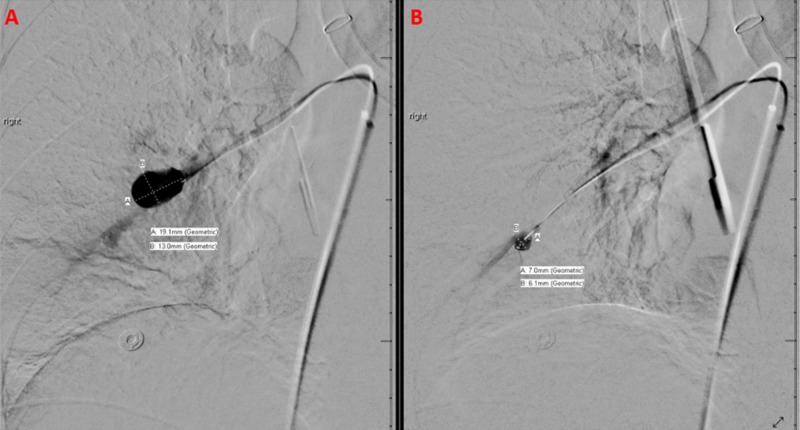
Selective Angiogram Demonstrating Two Separate Saccular Pulmonary Aneurysms The selective angiogram demonstrates two separate saccular aneurysms in the lateral pulmonary arterial segment of the right middle lobe. The left frame (A) shows the proximal-most saccular aneurysm, measuring 19 by 13 millimeters in diameter. The right frame (B) shows the distal aneurysm, measuring seven by six millimeters in diameter.

This artery was selectively catheterized with a microcatheter and microwire. A selective angiogram demonstrated two separate saccular aneurysms. The proximal aneurysm measured 13 by 19 mm, with the aneurysmal neck measuring 5 mm; the distal aneurysm measured seven by six millimeters, with the aneurysmal neck measuring 3 mm (Figure 2).

**Figure 3:**
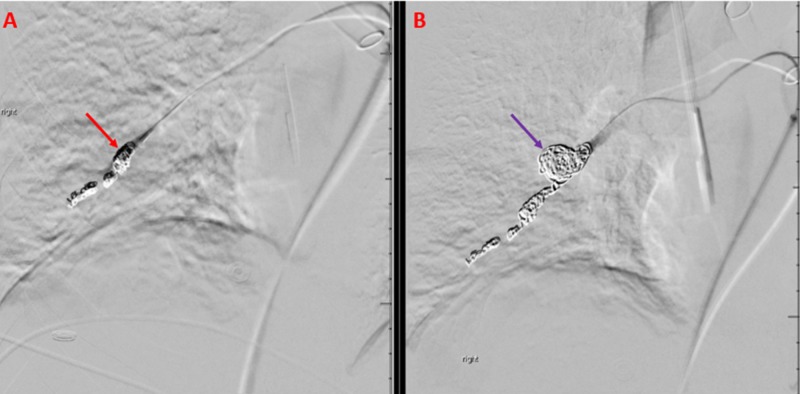
Post-Embolization Angiography Demonstrating Complete Obliteration of the Pulmonary Aneurysms The “sandwich technique” for aneurysm embolization was used to obliterate both proximal and distal pulmonary aneurysms. Various metallic coils were used for the embolization processes of both aneurysms. The left frame (A) shows the complete embolization of the distal aneurysm with metallic coils (red arrow). The right frame (B) shows the complete embolization of the proximal aneurysm with metallic coils (purple arrow).

Using the “sandwich technique” for aneurysm embolization, both the proximal and distal aneurysms were embolized by placing a total of 11 metallic coils [6]. Two Terumo® Azur CX coils (Terumo Medical Corporation, Somerset, NJ, US), six Boston Scientific® Interlock-18 coils (Boston Scientific, Marlborough, MA), and three Terumo® Microvention HydroFrame coils (Terumo Medical Corporation, Somerset, NJ, US), were used to embolize from distal to proximal, up to the origin of the vessels. Post-embolization angiography demonstrated complete obliteration of the aneurysms (Figure 3).

Finally, a Bard® Denali (C. R. Bard Incorporated, Murray Hill, NJ, US) infra-renal retrievable IVC filter was placed. The procedure was performed uneventfully without complications. The patient was observed in the hospital for four days post-procedurally and for treatment of the underlying Behcet’s disease. The patient was then discharged home in a favorable condition.

## Discussion

Treatment for PAAs occurring in the context of Behcet’s disease include medical, surgical, or endovascular management [7]. Surgical and endovascular management are required for symptomatic patients, as well as treatment-resistant and larger-sized PAAs [7-8]. Traditionally, surgical repair has been recommended for PAAs larger than six centimeters and in patients who are symptomatic, regardless of aneurysm size [8]. In the setting of the preceding patient, the patient presented with two PAAs that were less than six centimeters and was an appropriate candidate for interventional embolization. Though the patient was actively bleeding upon presentation, both surgical and interventional teams concluded that embolization would be appropriate, according to the state the patient was in. The increased conventional use of endovascular embolization has highlighted the advantages of treating PAAs outside the traditional guidelines, as represented in the preceding case.

The surgical management of PAAs includes aneurysmorrhaphy, lobectomy, bilobectomy, pleurectomy, aneurysmectomy, and pneumonectomy [2]. These procedures have been associated with high rates of morbidity and the recurrence of hemoptysis [2]. Complications such as perivascular leaks, anastomotic leaks, and graft thrombosis have also been reported [2]. Surgical lobectomies, in particular, cause an increased risk of developing recurrent PAAs or pseudoaneurysms, secondary to increased post-operative pulmonary pressures [9]. Specifically, surgical management has been challenging for patients with Behcet’s disease since their complete care often includes immunosuppressant medications, which can impede post-surgical healing. Furthermore, due to the high-risk of PAA rupture, surgical repair is not recommended as the standard of treatment for large pseudoaneurysms [9]. While surgery has been noted to be the indicated management in emergencies, the increased risk of complications makes an interventional endovascular approach more appropriate in such cases [4]. It has even been reported that endovascular intervention in certain patients, such as the one presented, has been recommended in the hemodynamically unstable. This has demonstrated that interventional management can salvage pulmonary function and improve prognosis over surgical options [4]. Specifically pertaining to the preceding case, it was determined that the management of the patient's sudden onset of hemoptysis was not emergent and did not require immediate surgical intervention, as professionally determined by both the surgical and interventional teams. This made it appropriate for the interventional team to perform an endovascular intervention, all while salvaging a larger portion of the patient's pulmonary function, which would have been otherwise lost during a surgical procedure.

As opposed to surgery, interventional embolization of multifocal PAAs and pseudoaneurysms has proven to be advantageous [10]. This method of management includes the use of glue embolization, balloon embolization, stent graft placement, or the use of coils [11]. These procedures have the advantage of decreased lethality and post-procedure complications. While the theoretical risk of aneurysmal rupture with endovascular procedures exists, no cases of such complications have been reported to date [2].

The less invasive nature of intravascular approaches makes them preferable to surgery when comparing the two methods of management [4]. While surgery has been the prior standard of care, the broadened conventionality of PAA embolization has rapidly developed. Though there are clear guidelines for certain patient presentations, it is important for interventionists and surgical teams to be interdisciplinary. For patients who have complex presentations and surgical or interventional management is not clear, both the surgical and interventional teams should interprofessionally aspire to collectively attain the best patient outcomes. As in the preceding patient presented, both surgical and interventional teams provided their professional insight that led to the appropriate interventional management of PAAs and the preservation of pulmonary function.

## Conclusions

It has been recently reported that various interventional embolization methods have been successfully utilized to treat PAAs over surgical management. While these patients would have formerly been treated surgically, current literature has shown increasing success in the use of endovascular embolization, even in patients that are symptomatic, hemodynamically unstable, or in states of decompensation. The practical use of endovascular embolization is becoming a favorable option for treating PAAs and has shown decreased morbidity and mortality over surgical management in a spectrum of etiologies.
